# Imbalance of Excitatory and Inhibitory Neurotransmitter Systems in Myalgic Encephalomyelitis/Chronic Fatigue Syndrome

**DOI:** 10.3390/ijms27094041

**Published:** 2026-04-30

**Authors:** Klaus J. Wirth, Carmen Scheibenbogen

**Affiliations:** 1 Mitodicure GmbH, 65830 Kriftel, Germany; 2 Institute of Medical Immunology, Charité—Universitätsmedizin Berlin, Corporate Member of Freie Universität Berlin and Humboldt Universität zu Berlin, 10117 Berlin, Germany

**Keywords:** Myalgic Encephalomyelitis/Chronic Fatigue Syndrome (ME/CFS), Long COVID, neurotransmitter imbalance, autonomic dysfunction, noradrenaline, GABA, glutamate, serotonin, hypersensitivities

## Abstract

Myalgic Encephalomyelitis/Chronic Fatigue Syndrome (ME/CFS) and post-COVID-19 syndrome share a symptom profile, including severe fatigue, cognitive dysfunction, exertional intolerance, sleep disturbances, hypervigilance, and the paradoxical state of being “wired but tired.” A well-established finding is sympathetic hyperactivity with reduced vagal tone, typically interpreted as autonomic nervous system dysfunction. Emerging evidence, however, suggests a broader disturbance across multiple neurotransmitter systems. This paper reviews current knowledge on neurotransmitter systems implicated in ME/CFS and Long COVID, focusing on potential mechanisms of dysregulation and their roles in disease pathology and symptom generation, as well as implications for treatment. In addition to abnormalities of the noradrenergic system, disturbances in serotonergic, GABAergic, and glutamatergic signaling have been reported. Contributing factors may include autoimmunity, neuroinflammation, gut dysbiosis, epigenetic influences, and stressors such as orthostatic intolerance, metabolic strain, and pain. A shift favoring excitatory over inhibitory neurotransmission can lead to excessive neural activation, autonomic dysfunction, sensory hypersensitivities, sleep disturbances, and cognitive impairment. Reduced GABAergic tone combined with increased glutamatergic and noradrenergic activity may elevate skeletal muscle tone, contributing to calcium overload, mitochondrial dysfunction, exertional intolerance, and post-exertional malaise. Various pharmacological treatments may partially rebalance these neurotransmitter systems, but limited efficacy highlights the need for systematic investigation and individualized strategies.

## 1. Introduction

Among the key symptoms of fatigue, disturbed cognitions, and exertional intolerance with post-exertional malaise (PEM), Myalgic Encephalomyelitis/Chronic Fatigue Syndrome (ME/CFS) patients commonly show sympathetic hyperactivity, sleep disturbances, non-restorative sleep and nocturnal hypervigilance and the feeling of overstimulation despite profound fatigue [1]. This paradox is described by “wired but tired”. Long COVID shares many symptoms and findings with ME/CFS and a subset of patients fulfill the diagnostic criteria for ME/CFS [2].

Sympathetic hyperactivity, reduced vagal tone, and an impaired sympathovagal balance have long been recognized, indicating disturbances in noradrenergic and cholinergic signaling [3,4,5,6,7,8,9,10,11,12,13,14]. In recent years, growing evidence suggests that additional neurotransmitter systems are also affected. In this paper, we review the current evidence of neurotransmitter disturbances in ME/CFS and Long COVID and compiled the findings. We provide evidence that the dysregulation of neurotransmitters may extend beyond the disturbed sympathetic–vagal balance and involve multiple neurotransmitter systems. This broader imbalance of neurotransmitters could favor excitatory over inhibitory signaling, causing CNS overstimulation that can contribute to autonomic dysfunction, sensory hypersensitivities, cognitive impairment, and skeletal muscle pathology. In the second part of this paper, we try to explain how these neurotransmitter dysregulations could drive disease pathology and explain core symptoms and complaints.

It is important to consider that autonomic dysfunction may arise, at least in part, as a consequence of the underlying neurotransmitter imbalance, while additional mechanisms are likely involved. Furthermore, once fully developed, ME/CFS may further exacerbate these neurochemical disturbances. Physiologically, the interplay of excitatory and inhibitory systems determines overall activity levels, closely tied to the sleep–wake cycle. In ME/CFS, this state-dependent balance appears disturbed both during the day and at night.

Our deeper analysis of neurotransmitter disturbances is motivated by two findings unrelated to the sympathetic nervous system and its transmitter noradrenaline. First, a recent study identified several new autoantibodies, including one against serine/arginine repetitive matrix protein 3 (SRRM3), which is linked to the GABA system [15,16]. Notably, a single nucleotide variant in SRRM3 has been associated with ME/CFS, implicating dysfunctional SRRM3, which may disrupt GABA-related splicing and impair GABAergic signaling in disease pathophysiology [17] (Supplementary Table S1). Supporting this notion, a recent magnetic resonance study has reported elevated glutamate levels in ME/CFS, consistent with an imbalance of the glutamatergic–GABAergic neurotransmission [18]. Second, several studies have reported reduced tryptophan levels in ME/CFS and Long COVID, that could lower the availability of this precursor for the synthesis of serotonin. Together, these findings suggest that the GABAergic and serotonergic systems may also be disturbed.

Prompted by these insights, we systematically reviewed the literature for further potential disturbances in major neurotransmitter systems, focusing on excitatory (noradrenaline, histamine, glutamate, and dopamine) and inhibitory or modulatory neurotransmitters (GABA, serotonin, and vagal acetylcholine). We would like to emphasize that, apart from sympathetic hyperactivity, the evidence for disturbances of other neurotransmitters is still scarce and requires further research. Nevertheless, there are sufficient indications to seriously consider the possibility that an imbalance of the main neurotransmitter systems beyond the well-known disturbance of the sympathetic–parasympathetic axis may contribute to and potentially even play a causal role in the initiation of the disease.

Consistent with the assumptions of disturbed neurotransmitters, a range of pharmacological interventions targeting these neurotransmitters have been applied empirically in ME/CFS. Although no large-scale clinical trials have demonstrated efficacy, several drugs show beneficial effects in subsets of patients: clonidine and beta-blockers (noradrenaline modulation), vagal activation procedures and pyridostigmine (effects on vagal and sympathetic first ganglion), serotonin reuptake inhibitors (SSRIs), aripiprazole (presumably dopamine receptor modulation), antihistamines, dextromethorphan or memantine (NMDA-glutamate receptor modulation), and benzodiazepines (GABAergic activation), and presumably also low-dose naltrexone (LDN), as will be explained below. Collectively, these interventions seem to reflect a therapeutic strategy of rebalancing disturbed neurotransmitter systems, although these drugs are not administered with this explicit therapeutic concept.

The causes of these neurotransmitter disturbances may involve immune dysregulation (autoantibodies, neuroinflammation, and mast cell overactivity), genetic predisposition, epigenetic regulation, and gut dysbiosis, which will be discussed in the following sections on the single neurotransmitters. These factors may create a vulnerability to developing ME/CFS following infection. Once ME/CFS is fully established, it seems to further augment and aggravate the neurotransmitter disturbances and autonomic dysfunction. Table 1 gives an overview of the main neurotransmitters and their localization and function. We would like to emphasize that not all neurotransmitters need to be equally affected in every patient. While disturbances in the sympathetic–vagal axis of GABA and serotonin signaling appear relatively pronounced, other systems might also be altered, although the extent of their contribution is currently unclear. Nevertheless, together these disturbances could contribute to the overall imbalance of neurotransmitters in ME/CFS.

## 2. Disturbances of the Neurotransmitter Systems in ME/CFS

### 2.1. Noradrenergic System

Sympathetic hyperactivity, reflected by altered heart rate variability, has been shown in numerous publications in ME/CFS [3,4,5,6,7]. It is primarily driven by increased noradrenergic activity. Nocturnal hypervigilance, sleep disturbances, and the feeling of overstimulation may result from an overactive noradrenergic system, in which the locus coeruleus plays a central role. Another consistent observation is reduced vagal activity [3,8,9,10,11,12], indicating a clear imbalance between noradrenergic and cholinergic vagal signaling [13].

Autoimmunity appears to play a central role in a subset of ME/CFS patients, with autoantibodies reported against several receptors. The best-studied autoantibodies to date are those against β_2_AdR, with higher levels correlating with symptom severity and structural alterations in the central nervous system [19,20]. A recent study identified autoantibodies targeting, among others, the α1-adrenergic and α2C-adrenergic receptors [16]. Autoantibodies against the α1- and α2-receptors may contribute to orthostatic dysfunction, indirectly activating the noradrenergic system via orthostatic stress. In contrast, antibodies against the α2C-receptor could also directly increase noradrenergic activity by enhancing noradrenaline release from sympathetic neurons and nerve endings [21]. Indeed, antibody levels against a1- and a2-receptors were found to be significantly associated with the severity of autonomic dysfunction and fatigue [16,22]. In post-COVID-19 syndrome patients, autoantibodies against angiotensin II receptor type 1/2, against adrenoceptor beta 1/2, against muscarinic acetylcholine receptor M1/M3, and against C-X-C motif chemokine receptor 3 (CXCR3ab) were associated with altered parasympathetic and sympathetic tone [23].

Noradrenergic activity may also rise if catecholamine metabolism is impaired. Some studies showed that ME/CFS is associated with genetic variations in catechol-O-methyltransferase (COMT), the main enzymes degrading catecholamines [24,25], but could not be confirmed by others [26,27]. An epigenetic investigation, however, reported about double the DNA methylation in the MB-COMT promoter region seen in healthy controls, and this was considered an independent factor contributing to the symptoms and pathophysiology of ME/CFS with concomitant fibromyalgia [26]. Decreased expression of COMT would enhance the activity of both noradrenaline and dopamine (discussed below) and thus contribute to the neurotransmitter imbalance. These factors may represent risk conditions that, under stress, disturb the balance between excitatory and inhibitory neurotransmitter systems. An open question is whether DNA methylation of COMT is a primary or secondary event.

Stress is a major pathogenic factor in ME/CFS. Most patients suffer from orthostatic intolerance and reduced cerebral blood flow, and in Long COVID this has been shown to be prevalent already early in the disease course [28,29,30]. Orthostatic dysfunction causes orthostatic stress, further activating the sympathetic system. Additional stressors—metabolic strain from poor skeletal muscle energetic situation, respiratory stress, pain, and psychosocial stress—likely stimulate the system. The expected sympathetic hyperactivity, hyper-alert state, and nocturnal hypervigilance are consistent with enhanced excitatory noradrenergic influences. However, surprising findings require deeper consideration: A recent study reports elevated resting heart rates but diminished heart rate response during physical activity in individuals with post-COVID-19 ME/CFS symptoms [31]. In a group of patients with post-infection ME/CFS, DOPA and metabolites of dopamine and noradrenaline in cerebrospinal fluid were decreased [32]. Levels of noradrenaline and dopamine did not differ compared with healthy controls, while sympathetic tone was found raised in the same patients as in many other studies. Resting heart rate was found higher but peak heart rate was lower in the cardiopulmonary exercise test in this paper. A systematic review and meta-analysis found adrenergic dysfunction in patients with ME/CFS. The authors concluded that higher baseline adrenaline levels and atypical responses to exercise in ME/CFS indicate sympathetic dysfunction and adrenergic abnormalities [14]. There is no easy answer to these partly conflicting findings. A conclusion is that the noradrenergic system must be dysregulated. But how? High sympathetic tone with tachycardia as in ME/CFS cannot be reconciled with cerebral catecholamine depletion. Experimental catecholamine depletion by reserpine causes among other effects, bradycardia, a decrease in blood pressure, hypothermia, and sedation [33]. Alpha2-adrenergic agonists like clonidine and dexmedetomidine that inhibit noradrenaline release by activating the presynaptic alpha2-adrenergic autoreceptor, cause bradycardia, and are even used for sedation clearly demonstrating the effects of decreased noradrenaline availability at its postsynaptic receptor [34]. ME/CFS patients cannot sleep, show tachycardia, often have subfebrile temperature, are usually not depressive, and are “wired”. These contradictory findings can be reconciled by the assumption that Locus Coeruleus is permanently overstimulated by stressors. Such permanent stimulation requires much energy; however, supply is impaired due to malperfusion and disturbed neurovascular coupling [35,36,37,38]. As a result, Locus Coeruleus may be unable to recover fully, leading to an inadequate sympathetic and adrenergic response for exercise and for orthostatic regulation. The possibility should also be considered that the ability of the noradrenergic and adrenergic system to appropriately adapt its activity to exercise and orthostatic regulation may further worsen over time and particularly with increasing disease severity. Orthostatic dysfunction, hypovolemia, and the poor energetic situation in skeletal muscle (see below) may even require a higher-than-normal level of sympathetic activation for everyday activities in ME/CFS. In patients with connective tissue abnormalities and dilated veins due to weakness of the vascular connective tissue, the noradrenergic stimulation required to maintain adequate cardiac preload when standing and to further increase it during exercise is likely higher than normal. Contractile stimulation, however, may even be lower due to a disturbed noradrenergic system (too high at rest—too low on demand). The finding that patients with ME/CFS exhibit normal hemodynamics at rest but a marked reduction in cardiac output during exercise, as demonstrated by invasive CPET, supports this interpretation [39]. However, a potential contribution of hypovolemia to the reduced cardiac output should also be considered.

In addition to impaired contraction of capacitance vessels due to insufficient α-adrenergic stimulation—limiting cardiac preload during orthostatic stress and exercise—β-adrenergic mechanisms may also be involved. In ME/CFS, β_2_AdR function may be compromised by three mechanisms: β_2_AdRs are particularly prone to desensitization, as shown in heart failure and experimental studies [40,41]. (1). In ME/CFS, sustained sympathetic overactivity may promote β_2_AdR desensitization. (2) β_2_AdR autoantibodies may further impair receptor function. (3) Chronic overstimulation may lead to functional “exhaustion” of the adrenergic system, thereby limiting its capacity to adequately activate β_2_AdR during exercise. Because these receptors are already compromised by the two mechanisms mentioned, an even greater level of adrenergic stimulation would be required to achieve normal function for exercise, but adrenergic stimulation may be lower due to “exhaustion”. Because β_2_AdRs increase blood flow to skeletal muscle, brain, and heart and mediate bronchodilation, their dysfunction limits exercise capacity.

Proper function of ß2AdR is not only important with respect to adequate perfusion and respiration but also for skeletal muscle function. In skeletal muscle not only perfusion, but, probably even more importantly, stimulation of the Na^+^/K^+^-ATPase may be impaired by a disturbed ß2AdR function [42,43,44]. This is because during exercise the stimuli of this ion transporter are ß2AdR and calcitonin-gene-related peptide (CGRP) released from sensory nerve fibers. CGRP, physiologically released from small sensory nerve fibers, is likely diminished as soon as small fiber neuropathy has developed. The functional consequences of a disturbed overall function of ß2AdR for skeletal muscle pathophysiology and exercise intolerance will be highlighted below in a separate section.

ß2AdRs should be more disturbed than ß1AdRs due to their high sensitivity to desensitization, but an impaired maximal stimulatory capacity of noradrenergic output as argued above during exercise would also affect and impair the ß1AdR component. What are the effects of these disturbances on the heart? Chronotropic incompetence during exercise has been reported which can be fully explained by deficient ß-adrenergic stimulation as both ß-adrenergic receptor subtypes exert chronotropic effects [45]. Implicating deficient ß-adrenergic stimulation for causing chronotropic incompetence automatically leads to the assumption of an impaired inotropy and lusitropy (impaired diastolic relaxation for adequate cardiac filling) because these actions are also mediated via ßAdR-mediated rise in cAMP. A paper reported shortening of QTc interval in ME/CFS patients [46], which can only be attributed to dysfunction of ß2AdR because the cardiac action potential is physiologically shortened by ß1AdR activation but prolonged by ß2AdR activation [47,48]. This means both ß-adrenergic receptor subtypes have opposing effects on the action potential duration (reflected by the QT-time in the ECG). Shortening of the action potential also has a mild negative inotropic effect forcing down the calcium current and thereby calcium influx in the heart beyond the direct effect of diminished cAMP on the different mechanisms involved in cardiac calcium dynamics.

Pyridostigmine, which has been shown to improve orthostatic dysregulation and muscular fatigue in ME/CFS [49,50], can stimulate and amplify sympathetic tone in ME/CFS: The first ganglia of both the sympathetic and the parasympathetic system are cholinergic (nicotinic). Raising acetylcholine by inhibition of degradation by pyridostigmine theoretically can raise the activity of both arms of the autonomic nervous system. Since inhibition of cholinesterase is an amplifying mechanism, it will particularly raise the activity of the system that is already centrally activated. It means that, when sympathetic drive starts for exercise or for orthostatic regulation, pyridostigmine preferentially amplifies sympathetic tone. By contrast, when central vagal tone is predominant at rest, the vagus nerve becomes more stimulated. Thus, pyridostigmine can theoretically improve the situation during both exercise and recovery. Pyridostigmine, which does not penetrate the blood–brain barrier, may have other favorable effects via enhanced acetylcholine concentrations at the vagal nerve terminals and as a neuromuscular transmitter that also activates the Na^+^/K^+^-ATPase at rest (not during exercise) [42]. Additionally, the effects of acetylcholine via the α7-nicotinic (α7-nAChR)- and M3-cholinergic receptors subtype on endothelia cells are considered beneficial [51].

Stress-induced sympathetic activation typically reduces vagal tone. Further impairment may arise from gut dysbiosis, which disturbs the gut–brain axis [52,53,54,55]. This axis largely depends on serotonin release from enterochromaffin cells, linking vagal activity to serotonergic signaling [56,57]. Thus, a disturbed gut microbiome might therefore weaken vagal activity. Whether efferent vagal output e.g., to the heart and cardiovascular system, is influenced by reduced serotonergic afferent vagal input from the gut remains to be demonstrated, however. Therapeutic interventions that stimulate the vagus nerve or enhance acetylcholine release may help improve the vagal tone.

Altogether, there is strong evidence that the noradrenergic system is dysregulated—characterized by elevated activity at rest, but likely insufficient activation during exertion and possibly impaired orthostatic regulation due to exhaustion.

### 2.2. Serotonin

Serotonin, a modulatory neurotransmitter, is likely to play a role in ME/CFS, and there is some evidence suggesting that its levels are reduced in the central nervous system. Reduced tryptophan availability—caused by gut dysbiosis [52,53,54,55] and diversion of tryptophan into the kynurenine pathway via increased IDO2 activity—is assumed to cause decreased serotonin synthesis [58,59]. Several studies show that the amount of tryptophan in blood is decreased in post-COVID-19 syndrome patients [60,61,62]. Another possible mechanism lowering the level of the serotonin precursor tryptophan is an increased activity by tryptophan-catabolizing enzyme indoleamine 2,3-dioxygenase (IDO1), which is typically induced by interferon in viral infections [58].

Levels of serotonin or its metabolite 5-methoxytryptamin in blood are found decreased or unchanged in post-COVID-19 and ME/CFS patients [63,64,65,66]. In another ME/CFS study, plasma serotonin was not altered in women but significantly reduced in men. The ratio of tryptophan to serotonin was elevated in patients suggesting lower serotonin levels according to the authors [67]. Therefore, serum serotonin does not seem to be a reliable biomarker. Furthermore, serotonin does not cross the blood–brain barrier to act as a neurotransmitter. In the brain, serotonin is generated from tryptophan, such that tryptophan levels in blood, which are diminished, may be more relevant as a proxy. Serotonin’s role in stimulating afferent vagal activity in the gut via chromaffin cells through gut–brain interactions may also affect vagal function in Long COVID and ME/CFS [56,57].

Clinical observations suggest that serotonin reuptake inhibitors (SSRIs) can provide some symptomatic relief, supporting the hypothesis of serotoninergic dysregulation in post-COVID-19 patients [68]. SSRIs were shown to improve fatigue, cognitive impairment, and hypersensitivity in ME/CFS. Raising serotonin is probably not beneficial in all respects: Post-COVID-19 syndrome patients are more sensitive to side effects of SSRIs than other patients [68]. Serotonin, particularly in the spinal cord, can raise motor neuron excitability and output via the 5-HT2 receptor subtype [69,70,71]. In severe ME/CFS, skeletal muscle membrane is most likely depolarized and therefore hyperexcitable [72] (discussion below) and serotonin may have the potential to worsen this aspect of muscle symptomatology. In summary, there is evidence suggesting a deficit of serotonin in the central nervous system in ME/CFS.

### 2.3. GABA

Several findings suggest an impairment of the GABAergic system in ME/CFS, which is the most important inhibitory system in the brain. This impairment may result from TRPM3 dysfunction as well as from genetic or autoimmune disturbances of SRRM3, a protein involved in GABA signaling. Both a SNP and autoantibodies against SRRM3 have been found in subsets of patients and linked to ME/CFS, raising the suspicion that GABAergic signaling could be disturbed [16,17], (Supplementary Table S1). A pilot study also reported reduced urinary GABA levels [73]. TRPM3 dysfunction has been documented in natural killer cells, and low-dose naltrexone (LDN) has been shown to restore ion channel function [74,75,76]. Since TRPM3 is also expressed in sensory Aδ- and C-fibers and in neurons of the CNS, where it is involved in the secretion of GABA [77], LDN may indirectly improve GABAergic signaling in the brain by improving TRPM3 function [78]. This also fits into the concept of neurotransmitter rebalancing.

GABA plays a critical role not only in sleep regulation but also in skeletal muscle tone, where it exerts a relaxing effect [79,80]. Conversely, noradrenergic and glutamatergic activity increases muscle tone. Thus, an imbalance between noradrenaline, glutamate, and GABA at the muscular level may contribute to ME/CFS muscle pathology [72]. Additionally, GABA normally counterbalances excitatory glutamate, suggesting that impaired GABA function could explain observed increases in glutamate (see below in the section on glutamate). The pathophysiological consequences of an elevated skeletal muscle tone as a consequence of an imbalance of these neurotransmitters involved in setting the muscle tone will be discussed below. Benzodiazepines (e.g., lorazepam), which enhance GABA receptor activity, are one of the most effective treatments for fatigue and sensory hypersensitivity in ME/CFS; however, their clinical use is limited by the risk of dependency. In summary, there is strong evidence of diminished GABAergic system activity in ME/CFS.

### 2.4. Glutamate

Glutamate, an excitatory neurotransmitter, is likely to play a role in ME/CFS. There is some evidence suggesting that this neurotransmitter system is overactive in ME/CFS. A recent MRI study reports elevated brain level of glutamate and *N*-acetyl-aspartate in the brains of Long COVID and ME/CFS patients [18]. Glutamate is the most important excitatory neurotransmitter in the brain. Since GABAergic signaling normally inhibits glutamatergic activity, reduced GABAergic function could contribute to this increase (see section on GABA). An increased density of AMPA-type glutamate receptors on the post-synaptic neural cell surface was found to be associated with cognitive impairment in Long COVID in a recent study using [^11^C]K-2 PET imaging [81], demonstrating a possible altered glutamatergic receptor signaling, rather than altered transmitter levels itself. The data therefore suggests disturbance of the neurotransmitter systems in a broad sense like in the case of alpha2-C-adrenergic receptors.

There is increasing evidence suggesting a role of neuroinflammation in ME/CFS [82,83,84,85,86]. Low-grade neuroinflammation indicated by cytokines found in cerebrospinal fluid may act on astrocytes and influence the handling of the neurotransmitters glutamate and GABA [87]. Astrocytes take up 80% of the neuronally released glutamate but only 20% of the released GABA [88]. Both neurotransmitters are recycled via the astrocytes and returned to the neurons as glutamine, which, in turn, release glutamate and GABA. Cytokines, generated in the process of neuroinflammation, are supposed to reduce both glutamate and GABA uptake in astrocytes. Since astrocytic glutamate uptake is much higher than that of GABA, the cytokine-induced inhibition of neurotransmitter uptake in astrocytes should result in an imbalance in favor of glutamate over GABA. This would increase neuronal excitatory effects of glutamate in the brain [89].

Memantine and Dextromethorphan, which act as NMDA (glutamate) receptor antagonists, have been discussed as potential treatment, although data on their efficacy in ME/CFS are not available. In an open-label phase II study in patients with Long COVID, amantadine acting as an NMDA receptor antagonist, among other properties, was associated with an improvement in fatigue. In contrast, a placebo-controlled trial in patients with ME/CFS did not demonstrate a significant effect [90,91]. This discrepancy may in part be explained by poor tolerability of amantadine in ME/CFS patients, potentially related to its dopaminergic and anticholinergic properties. GABA has crucial inhibitory effects in the brain directly counteracting the excitatory effects of glutamate. The efficacy of benzodiazepines activating the GABA system indirectly supports the notion that dysregulation of the glutamate receptor plays an important role in disturbing the neurotransmitter balance. Altogether, there is evidence that the glutaminergic system is overactive in ME/CFS.

### 2.5. Glycine

In the central nervous system glycine acts as an inhibitory neurotransmitter in certain regions of the CNS (e.g., spinal cord, brainstem). In addition, as a co-agonist at the NMDA receptor, it is involved in glutamatergic signal transmission (excitatory or modulatory). Glycine can be transported via the blood-brain-barrier by amino acid transporters (ASC transporter), but quantitatively its generation as a local neurotransmitter in the CNS from serine via the one-carbon metabolism seems to be by far more important [92]. Serine and its derivatives sarcosine were found elevated in the cerebrospinal fluid of ME/CFS patients with a decrease in 5-methyltetrahydrofolate (5MTHF) suggesting general dysfunction of folate and one-carbon metabolism in ME/CFS [93]. Speculatively, this could reduce the availability of glycine as a local neurotransmitter in the CNS.

### 2.6. Dopamine

Dopaminergic activity may also be enhanced due to genetic or more likely epigenetic changes of COMT, such as altered DNA methylation, as explained above [26], reducing dopamine breakdown and raising its levels, but direct evidence is missing. Clinically, low-dose aripiprazole, a dopamine receptor modulator, has shown some benefit [94]. Its use at low doses is consistent with a broader therapeutic concept of neurotransmitter rebalancing.

### 2.7. Histamine

Histamine is an excitatory neurotransmitter in the brain. Many Long COVID and ME/CFS patients show increased mast cell activity. Peripherally, histamine likely contributes to pathophysiology through vascular effects: (i) inadequate arterial dilation causing blood diversion (“steal phenomenon”), and (ii) venous dilation via H_2_-receptors reducing venous contractility and preload (venous pooling) [95]. Hypovolemia due to vascular leakage may further aggravate these effects. As histamine is unable to cross an intact blood–brain barrier, mast cells as resident immune cells in the brain must be considered a potential pathological source of histamine within the central nervous system. Centrally, histamine functions as an excitatory neurotransmitter, especially in the tuberomammillary nucleus (TMN) of the hypothalamus [96], suggesting a potential role in hyperexcitability. Whether histamine acting as a central excitatory transmitter contributes to the disease remains to be investigated. An open question is whether a dysfunctional blood–brain barrier could allow histamine to enter the brain. A disturbance of the blood–brain barrier has been proposed in ME/CFS and Long COVID [83,97]. Among the antihistamines, brain-penetrating drugs include diphenhydramine as a H_1_-histamine receptor antagonist and cimetidine blocking H_2_-receptors.

### 2.8. Summary of the Neurotransmitter Disturbances

We have compiled the available information on potential disturbances of neurotransmitters in ME/CFS beyond the well-known dysregulation of the sympathetic and vagal system. There are preliminary data supporting the notion that additional neurotransmitter systems may also be affected, including glutamate, serotonin, and GABA, whereas for histamine, glycine, and dopamine such indications remain hypothetical.

**Figure 1:**
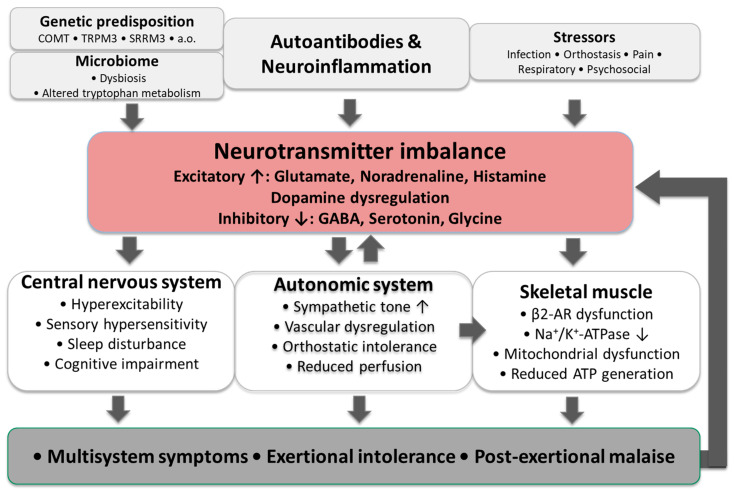
Proposed multifactorial model linking immune dysregulation, genetic susceptibility, microbiome alterations, and stressors to neurotransmitter imbalance, CNS symptoms, autonomic dysfunction, impaired bioenergetics, and resulting multisystem symptoms, including post-exertional malaise.

When these disturbances are considered in a context, they suggest a predominance of excitatory over inhibitory neurotransmission, leading to central nervous system overactivation that may underlie sympathetic hyperactivity, hypersensitivity, and skeletal muscle pathology, as illustrated in Figure 1.

## 3. Role of Neurotransmitter Imbalance in the Pathophysiology of ME/CFS and Their Potential Contribution to Typical Symptoms

After presenting the results of our search suggesting a predominance of excitatory over inhibitory transmitters in ME/CFS, we aim to explain how this neurotransmitter imbalance could contribute to the typical symptomatology of ME/CFS. These considerations raise the possibility that ME/CFS symptoms are also influenced to a relevant extent by disturbed neurotransmitter regulation. The term “neurotransmitter imbalance” implies that it is most likely not a single neurotransmitter disturbance, but rather the synergistic interaction of several neurotransmitter disturbances that gives rise to the ME/CFS symptomatology. As outlined earlier, this imbalance could be driven by autoantibodies, mast cell overactivity, low level neuroinflammation, tryptophan deficiency, genetic predisposition or epigenetic changes, and exposure to various stressors, with orthostatic stress likely playing a particularly important role.

Autoantibodies against peripheral neurotransmitter receptors and other structures can certainly play an important role in the disease pathology in ME/CFS such as antibodies against β_2_AdR, alpha1-adrenergic receptors, M3-cholinergic receptors, and the potassium-dependent sodium-calcium-exchanger NCKX3/ SLC24A3, to name a few [16], but the disturbance of centrally acting neurotransmitters may be particularly relevant for the severity of the neurological symptoms, sleep disturbances, and hypersensitivities and may also influence skeletal muscle pathophysiology, as will be outlined in the following sections.

### 3.1. Sleep Disturbances, Cognitive Impairment, and Sensory Hypersensitivities

A predominance of excitatory over inhibitory neurotransmitter systems will lead to sleep disturbances, nocturnal hypervigilance, and brain overstimulation. This obviously hyper-alert state is associated with overactivation of brain regions that could also cause sensory and perceptual disturbances and hypersensitivities. As explained in a recent publication [98], these disturbances may be due to an overactivation of the anterior insula within the salience network, leading to excessive sensitivities. The authors further explain that this is likely related to the low excitability threshold of the anterior insula, with excessive stimuli leading to overmobilization of responses in non-threatening situations [99,100]. In support of these notions, increased activation of the salience network was found in patients with ME/CFS [101]. Therefore, we consider the possibility that the symptoms involving sensitivity to lights, noises, and smells could be the result of an overactivation of the anterior insula by excitatory neurotransmitters while inhibitory influences are impaired. Thus, brain and particularly insula overactivation could lead to enhanced stimulus uptake in all sensory organs, while stimulus processing may be impaired because of a reduced global and local cerebral blood flow [35,36,37,38]. The strongly enhanced incoming sensory information would not be appropriately processed to finally judge them realistically as non-threatening, which could further enhance the hyper-alert state.

Overstimulation of the anterior insula can contribute to pain as a hypersensitivity phenomenon by enhancing the subjective intensity and affective perception of incoming afferent algesic signals [102]. We assume that pain in ME/CFS arises from an interaction between central pain processing and peripheral algesic factors. The latter could include injury to skeletal muscles, muscle tension and cramps, energetic disturbances in skeletal muscle potentially leading to the compensatory release of vasoactive mediators with algesic and hyperalgesic properties, small fiber neuropathy, gastrointestinal and pelvic disturbances, and connective tissue disorders—laxity of ligaments and joints and craniocervical instability [103,104,105,106].

Elevated neuronal activity, driven by a predominance of excitatory over inhibitory neurotransmission within the CNS, increases energy demand; however, cerebral energy supply is compromised by both reduced large-artery blood flow and impaired neurovascular coupling. [35,36,37,38]. Broad brain overactivation also diminishes the ability to concentrate and focus on a single demanding cognitive task. Combined with reduced cerebral perfusion and severely disturbed sleep, these disturbances could account for the cognitive impairments commonly observed in patients as explained previously [107]. Reduced ATP-to-phosphocreatine (PCr) ratio in a neuropsychiatric post-COVID patient was observed via ^31^P magnetic resonance spectroscopy in the cingulate cortex, indicating impaired brain energy metabolism, and lower ATP/PCr ratios specifically correlated with poorer cognitive performance [108]. Additional factors could worsen these disturbances: blood–brain barrier permeability appears to be increased, and intracranial pressure may be slightly elevated [104,109]. An energy deficit, chronic brain overstimulation without sufficient recovery, and insomnia together offer a plausible explanation for mental fatigue in ME/CFS.

### 3.2. Skeletal Muscle Pathophysiology

Enhanced noradrenergic and particularly glutamatergic activity, combined with reduced GABAergic signaling, increases skeletal muscle tone [110]. As we have described in a recent publication, skeletal muscle membrane—particularly in severely affected patients—appears to be in a state of depolarization, likely reflecting disturbed electrophysiology due to insufficient activity of the Na^+^/K^+^-ATPase required to maintain the negative resting membrane potential [72]. As the resting membrane potential then gets closer to the activation threshold causing hyperexcitability, increased central muscle tone due to a predominance of excitatory over inhibitory neurotransmitters involved in skeletal muscle tone regulation could therefore easily trigger abnormal excitations. These inappropriate excitations may manifest clinically as fasciculations and cramps and lead to sodium influx and potassium efflux, thereby further increasing the workload of the already impaired Na^+^/K^+^-ATPase. As a result, Na^+^/K^+^-ATPase can no longer restore the physiological membrane potential; depolarization worsens, calcium overload ensues, and mitochondria can be damaged. Mechanistically this involves reversal of the Na^+^/Ca^2+^ exchanger (NCX), which begins importing calcium instead of exporting it in the setting of elevated intracellular sodium and a more positive membrane potential. High stress levels, finally mediated by the excitatory neurotransmitters, additionally promote vasoconstriction and desensitize β_2_-adrenergic receptors. These receptors, together with CGRP during exercise, are the only hormonal activators of the Na^+^/K^+^-ATPase. Its insufficient stimulation contributes to intracellular sodium accumulation and calcium overload and subsequent mitochondrial and myocyte injury. Mitochondrial dysfunction, together with disturbances of perfusion and diffusion, impairs skeletal muscle energetics and can explain physical fatigue [111].

## 4. Why Current Treatments Targeting Neurotransmitter Imbalance Show Limited Efficacy in ME/CFS

At present, no treatment for ME/CFS provides satisfactory improvement, let alone disease remission. Several factors may explain the limited efficacy of currently available therapeutic approaches. Once ME/CFS is established, the dominant pathophysiological mechanisms appear to shift from early neurotransmitter imbalances toward self-sustaining pathomechanisms, including mitochondrial dysfunction, persistent skeletal muscle depolarization, oxidative stress, and impaired tissue perfusion. These processes mutually reinforce one another through complex feedback loops involving sympathetic overactivity, hypovolemia, inflammation, and chronic stress responses. As a result, cardinal clinical symptoms such as exercise intolerance and PEM become largely independent of the original disease trigger.

The effectiveness of pharmacological interventions targeting neurotransmitter systems is further constrained by substantial interindividual variability in the specific neurotransmitters affected, as well as by the fact that multiple physiological systems can be disturbed simultaneously. This limitation is particularly evident in patients with clear autoimmune signatures, with autoantibodies directed against multiple targets and receptors, often extending beyond classical neurotransmitter-related structures to include peripheral and non-neuronal structures. In such cases, interventions aimed at only one or two neurotransmitter systems may be insufficient or poorly aligned with the patient’s dominant pathomechanisms. For these patients, therapeutic strategies targeting autoimmune mechanisms may hold greater promise.

More effective rebalancing approaches may require carefully titrated combinations of drugs that address multiple neurotransmitter systems as well as central regulation of muscle tone, potentially including antihistamines. However, poor drug tolerability and marked interindividual variability pose major challenges to combination therapies. These factors necessitate very low starting doses, slow and cautious titration, and a high degree of clinical patience. Additionally, many patients appear to rely on elevated sympathetic activation as a compensatory mechanism to counteract metabolic fatigue and orthostatic dysfunction. While this persistent sympathetic overactivation may offer short-term functional compensation, it likely contributes to disease perpetuation and worsening over time. Consequently, in some patients, reducing sympathetic overshoot with sympatholytic agents may be beneficial, whereas in others it may aggravate symptoms and functional impairment.

## 5. Conclusions

Growing evidence indicates that, in addition to the noradrenergic system, several other neurotransmitter systems—particularly glutamate, serotonin and GABA—are dysregulated in ME/CFS. This imbalance, characterized by excessive excitatory relative to inhibitory signaling, may drive neural overactivation and autonomic dysfunction. These disturbances may cause key neurological symptoms and contribute to skeletal-muscle dysfunction that manifests as exercise intolerance, PEM, fasciculations, and cramps. The aim of future research should be to systematically investigate which neurotransmitter systems are affected across the ME/CFS patient population and to determine whether interindividual differences exist. Assuming substantial heterogeneity exists, a long-term objective should be to identify each patient’s specific neurotransmitter abnormalities to enable optimally individualized, targeted rebalancing therapies.

## Figures and Tables

**Table 1:** Overview of neurotransmitters, their receptors, main locations, and key functions.

Neurotransmitter	Effect	Receptors	Main Locations	Key Functions
Glutamate	Excitatory (main CNS excitatory transmitter)	Ionotropic: NMDA, AMPA, Kainate Metabotropic: mGluR (I–III)	Widely distributed in CNS (cortex, hippocampus, cerebellum)	Synaptic plasticity, learning and memory, motor control, sensory processing
Noradrenaline	Mainly excitatory	α_1_, α_2_, β_1_, β_2_, β_3_-adrenergic	Locus coeruleus, widespread CNS projections; peripheral sympathetic nerves	Arousal, attention, stress response, mood, blood pressure regulation
Acetylcholine (ACh)	Excitatory or inhibitory (receptor-dependent)	Nicotinic (ionotropic). Muscarinic (M1–M5) (metabotropic)	Neuromuscular junction, autonomic nervous system, basal forebrain	Muscle contraction, memory and learning, autonomic control
Histamine	Excitatory/ modulatory	H_1_, H_2_, H_3_, H_4_	Hypothalamus (tuberomammillary nucleus), peripheral	Wakefulness, attention, appetite control, immune responses
GABA (γ-aminobutyric acid)	Inhibitory	GABA-A (ionotropic, Cl^−^), GABA-B (metabotropic), GABA-C	Brain and spinal cord	Inhibitory control, anxiety regulation, sleep, muscle tone
Glycine	Inhibitory	Glycine receptor (ionotropic, Cl^−^), Co-agonist at NMDA receptors	Spinal cord, brainstem	Motor and sensory inhibition, reflex control, NMDA receptor modulation
Serotonin (5-HT)	Mostly inhibitory/ modulatory	5-HT_1–7_ (mostly metabotropic; 5-HT_3_ ionotropic)	Raphe nuclei, widespread CNS	Mood, sleep, appetite, pain modulation, emotional regulation

## Data Availability

No new data were created or analyzed in this study.
